# Antimicrobial potential of plant extracts from the Brazilian Cerrado

**DOI:** 10.1590/0103-6440202204705

**Published:** 2022-03-07

**Authors:** Luiz Evaristo Ricci Volpato, Paula Gabrielle de Castro Trigueiro, Andreza Maria Fabio Aranha, Ivana Maria Povoa Violante, Rafaela Alves da Silva, Rodrigo Cardoso de Oliveira

**Affiliations:** 1 Faculdade de Odontologia, Universidade de Cuiabá, Mato Grosso, Brazil.; 2 Departamento de Farmácia, Universidade de Cuiabá, Mato Grosso, Brazil.; 3 Centro Integrado de Pesquisas I e II, Faculdade de Odontologia de Bauru, Universidade de São Paulo, São Paulo, Brazil.; 4 Faculdade de Odontologia de Bauru, Universidade de São Paulo, São Paulo, Brazil

**Keywords:** Aggregatibacter actinomycetemcomitans, Streptococcus mutans, Enterococcus faecalis, Staphylococcus aureus, Escherichia Coli, Candida

## Abstract

Bacteria are related do different oral diseases, such as dental caries and periodontal disease. Therefore, the control or/and eradication of microorganisms and their by-products is primordial for the success of their treatment. An alternative for decrease bacterial load is the use of plant extracts used in popular medicine. The cytotoxicity and antimicrobial action of extracts of *Cariniana rubra Gardiner ex Miers*, *Senna martiniana*, *Anadenanthera colubrina* (*Vell.*) *Brenan* and *Spiranthera odoratissima St. Hil*. against strains of *Streptococcus mutans*, *Enterococcus faecalis*, *Staphylococcus aureus*, *Escherichia coli*, *Aggregatibacter actinomyces- tencomitans* and *Candida albicans* were investigated. Cytotoxicity was assessed at concentrations of 1, 10, 40, 80, 100 and 1000 μg/mL by means of the MTT test and compared to a control group with untreated cells. Those with acceptable cytotoxicity had the antimicrobial action measured by the XTT test. As a positive control, sodium hypochlorite was used. *Cariniana rubra Gardiner ex Miers* had the highest citototoxicity results while *Spiranthera odoratissima St. Hil*. had the best results, but all extracts showed acceptable cytotoxicity at different concentrations. The plant extracts showed higher activity against *A. actinomycetencomitans*: *Anadenanthera columbrina* (*Vell*.) *Brenan* (80.52%) at 40 μg/mL, *Spiranthera odoratissima St. Hil* (78.48%) in 1 μg/mL, *Senna martiniana* (73.28%) in the concentration of 40 μg/mL and *Cariniana rubra Gardiner ex Miers* (70.50%) in 10 μg/mL. All extracts analyzed showed acceptable cytotoxicity at different concentrations and were promising for inhibition of the pathogenic microorganisms studied.

## Resumo

Bactérias estão relacionadas a diferentes doenças bucais, como a cárie dentária e a doença periodontal. Assim, o controle e/ou erradicação de microrganismos e seus subprodutos é primordial para o sucesso dos tratamentos. Uma alternativa para diminuir a carga bacteriana é a utilização de extratos vegetais utilizados na medicina popular. A citotoxicidade e ação antimicrobiana de extratos de *Cariniana rubra Gardinerex Miers*, *Senna martiniana* H.S. Irwin & Barneby, *Anadenanthera colubrina* (*Vell*.) *Brenan* e *Spiranthera odoratissima St. Hil*. contra cepas de *Streptococcus mutans*, *Enterococcusfaecalis*, *Staphylococcus aureus*, *Escherichia coli*, *Agartibacter actinomycetencomitans* e *Candida albicans* foram investigados. A citotoxicidade foi avaliada nas concentrações de 1, 10, 40, 80, 100 e 1000 μg/mL por meio do teste MTT. Aqueles com citotoxicidade aceitável tiveram a ação antimicrobiana medida pelo teste XTT. *Cariniana rubra Gardinerex Miers* apresentou os maiores resultados de citototoxicidade, enquanto *Spiranthera odoratissima St. Hil*. obteve os melhores resultados, mas todos os extratos apresentaram citotoxicidade aceitável em diferentes concentrações. Os extratos vegetais apresentaram maior atividade contra *A. actinomycetencomitans*: *Anadenanthera columbrina* (*Vell*.) *Brenan* (80,52%) a 40 μg/mL, *Spiranthera odoratissima St. Hil* (78,48%) em 1 μg/mL, *Senna martiniana* H.S. Irwin & Barneby (73,28%) na concentração de 40 μg/mL e *Cariniana rubra Gardinerex Miers* (70,50%) em 10 μg/mL. Todos os extratos analisados apresentaram citotoxicidade aceitável em diferentes concentrações e foram promissores na inibição dos microrganismos patogênicos estudados.

## Introduction

The oral cavity is heavily colonized by hundreds of microorganisms ^1^. Differently from the commensal microbiota found at other body sites, which typically live in harmony with the host, oral cavity’s normal microbiota is associated to the occurrence of common diseases (dental caries and the periodontal diseases), especially under the influence of different host and environmental factors ^2^. Amongst the microorganisms frequently isolated from periodontal and/or endodontic lesions are *Candida albicans, Aggregatibacter actinomycetencomitans*, *Escherichia coli*, *Enterococcus faecalis, Streptococcus mutans* and *Staphylococcus aureus* ^3^.

Given the constant presence of diseases in the oral cavity, antimicrobial resistance represents a serious threat to the effective treatment of an increasing range of infections caused by bacteria, fungi and viruses worldwide, being a challenge for researchers and clinicians, especially in immunocompromised patients. Because of this scenario, researches are being carried out with the objective of obtaining new active compounds ^4^ ^,^ ^5^.

Since the dawn of humanity, nature has been an important source of antimicrobial agents ^6^ for the treatment of various diseases ^7^. Traditionally used in popular culture, especially in populations of developing nations, plants are also raw material for the preparation of herbal medicines or extraction of chemical compounds with therapeutic activity ^7^ ^,^ ^8^. The main advantages of medicinal plants are their perceived effectiveness, low incidence of adverse effects, low cost and accessibility ^4^ ^,^ ^5^ ^,^ ^6^ ^,^ ^7^ ^,^ ^8^. Researches carried out with medicinal plants are very important to confirm their safety and effectiveness ^7^ ^,^ ^9^.

Brazil is an important and promising source due to its great biodiversity, with more than 45,000 species of plants, comprising 20 to 22% of the total number of plant species in the world, having a wide variety of ecosystems ^7^. The *cerrado*, known as the tropical savanna with the greatest biological diversity in the world, has many medicinal plants that have not yet been investigated. This opens the opportunity to discover and develop new products in order to interfere in pathological processes ^7^.

From the results of previous studies and reports of popular use of plant extracts from the *cerrado* with antimicrobial purposes ^5^ ^,^ ^10^ ^,^ ^11^, specific extracts were selected for analysis. Thus, this work seeks to evaluate whether the plant extracts of *Cariniana rubra Gardiner ex Miers*, *Senna martiniana*, *Anadenanthera colubrina (Vell.) Brenan* and *Spiranthera odoratissima St. Hil*. are cytotoxic and have antimicrobial action against the pathogens *S. mutans*, *E. faecalis*, *S. aureus*, *E. coli*, *A. actinomycetemcomitans* and *C. albicans*.

## Materials and methods

### Preparation of extracts

The extracts were obtained at the Natural Products Center of the Faculty of Pharmacy, University of Cuiabá, Cuiabá, MT, Brazil. The specific parts of the plant species of *Cariniana rubra Gardiner ex Miers*, *Senna martiniana* H.S. Irwin & Barneby, *Anadenanthera colubrina* (*Vell*.) *Brenan* and *Spiranthera odoratissima St. Hil* were collected in the Rio Manso Region, in the municipality of Chapada dos Guimarães, MT, Brazil, in the dry season, having as coordinates: altitude 15°15'16''S and longitude 55°43'34''W. The material was herborized by the conventional method, which involves pressing, drying in an oven and fixation on cardboard, accompanied by catalog sheets containing the specimen's particular data. The exsiccates are deposited in the Central Herbarium of the Federal University of Mato Grosso (UFMT), where botanical identification was carried out using specific keys to identify the family, genus and plant species (Table 1). All were washed with running water and dried in a circulating air oven at 40°C, one week for leaves and two weeks for barks. Subsequently, crushing and spraying were carried out in an electric knife mill (Tecnal, Model 680, Piracicaba, SP, Brazil) and the material was macerated for seven days in ethyl alcohol 90% (1:2 p/v) at room temperature, during 7 days at 24 °C with daily homogenization. The filtrate was subjected to slow evaporation, under reduced pressure, at a temperature of 40°C, in a rotary evaporator (Fisaton, Model 802 - São Paulo, SP, Brazil) until the concentration of the extracts. The selected extracts are shown in Table 1. The plants studied are not included in the list of Brazilian plants threatened with extinction, therefore, their collection for purposes of scientific studies does not require prior authorization from the Brazilian Institute for the Environment and Renewable Natural Resources (IBAMA / MMA).

**Table 1:** Identification of species used in popular medicine, vernacular name and part of the plant used.

Identification	Family	Popular name	Used part	Exsiccate No.	Popular usage indication ^7^ ^,^ ^11^ ^,^ ^21^
*Cariniana rubra Gardinerex Miers*	*Lecythidaceae*	*Jequitibá*	Bast	39419	Used for the treatment of inflammatory diseases, especially throat diseases, oophoritis, venereal diseases, gastritis, hemorrhoids and tonsillitis.
*Senna martiniana* H.S. Irwin & Barneby	*Caesalpinioideae*	*Mata-pasto*	Leaves	23782	Used as a laxative, anti-allergy, anti-inflammatory, antioxidant, antibacterial, antimicrobial, analgesic, antiparasitic, insecticide, antitumor, hepatoprotective, antifungal and for skin disorders.
*Spiranthera odoratissima St. Hil.*	*Rutaceae*	*Manacá*	Leaves	23756	Used as a blood purgative, in the treatment of kidney and liver diseases, stomach pain, abdominal pain, headache and muscle pain, appetite stimulant, rheumatism, kidney infection, urinary retention, acne and boil.
*Anadenanthera colubrina* (*Vell.*) *Brenan*	*Mimosaceae*	*Angico*	Bast	23749	Used to treat respiratory diseases, inflammatory processes, diarrhea, cough, bronchitis, influenza, toothache, gastritis, pneumonia and colds.

### Cultivation and expansion of fibroblasts (NIH3T3)

NIH-3T3 fibroblasts (American Type Culture Collection - ATCC - mouse embryonic cell lineage) were cultured in DMEM culture medium supplemented with 10% fetal bovine serum (FBS) (Gibco® Invitrogen, Carlsbad, Ca, USA) and penicillin/streptomycin (100 IU/mL/100 μg/mL), and incubated in wet greenhouse (5% CO_2_/95% air, 37°C). Whenever the fibroblast culture reached subconfluence, it was trypsinized, that is, subjected to enzymatic treatment with 0.05% trypsin solution/0.02% EDTA (Sigma Aldrich, St Louis, MO, USA), and separated suspension for use in the cell viability assay.

### Cytotoxicity analysis

The cytotoxicity of the crude extracts was analyzed through the mitochondrial activity of the cells using the MTT reduction method (3-(4,5-dimethylthiazol-2-yl)-2,5-diphenyltetrazolium bromide). For this, 10^4^ cells/well were plated in 96-well plates. After incubation for 24 hours, the culture medium was replaced with DMEM medium supplemented by 10% fetal FBS containing the extracts (*Cariniana rubra Gardiner ex Miers*, *Senna martiniana* H.S. Irwin & Barneby, *Anadenanthera colubrina* (*Vell.*) *Brenan and Spirantheraodoratissima St. Hil*) in the concentrations 1, 10, 40, 80, 100 and 1000 µg/mL. Each plate was analyzed in an experimental time of 24 hours after adding the conditioned medium. To control group the cells were not treated (only DMEM medium supplemented by 10% fetal FBS). After each experimental period, the culture medium was removed, the cells were washed with Phosphate-Buffered Saline (PBS) and then the MTT reduction assay was performed.

In the experimental period (24h) the cells were washed with PBS, then the cells were incubated in a solution of 1 mg of MTT to 1 mL of DMEM without FBS, this solution was prepared at the time of use and was filtered in Millipore filter (0.22 µm) before being added to the plates. After this procedure, the plates were left for 4 hours at 37°C; then the solution was removed, the insoluble pigment reduced intracellularly was extracted in 150µL of dimethyl sulfoxide (DMSO) and left at room temperature for 30 minutes. Subsequently, absorbance was measured at 570 nm (Synergy MX Monochromator-based Biotek, Kyoto, Japan). The tests were performed in triplicates and the results were presented by the average of the values. The percentage of cell viability was determined by the following formula:

$$\%\;Cytotoxicity\;analysis\;=\;\frac{Average\;absorbance\;of\;test\;wells\;}{Average\;absorbance\;of\;control\;wells\;(Medium)}\;x\;100$$

### Evaluation of microbial metabolism

The antimicrobial effect of the extracts was analyzed only at the concentrations in which the cytotoxicity was acceptable by means of the colorimetric tetrazolium salt reduction assay - XTT (2,3-Bis (2-Methoxy-4-Nitro-5-Sulfophenyl) -5 - [(Phenyl-Amino) Carbonyl] - 2H-Tetrazolium Hydroxide - Sigma Aldrich Inc., St Louis, MO, USA). The antimicrobial activity of seven strains (*C. albicans* ATCC 90028, *C. albicans* SC 5314, *E. coli* O: 124, *S. mutans* ATCC 700610, *S. aureus* ATCC 6538, *A. actinomycetemcomitans* and *E. faecalis* ATCC 4083) was evaluated following protocols described previously ^12^. This determination was made after 24h of incubation at 37°C with the media conditioned by the extracts ^13^. For the use of XTT, a salt solution was previously prepared at a concentration of 1 mg/mL with Milli-Q water (Millipore Ind. and Com. Ltd, Barueri, SP, Brazil), being sterilized by vacuum filtration (PES 70 mm Diameter Membrane, 0.22 μm pore size, TPP®, Techno Plastic Products, Trasadingen, Switzerland). The XTT solution was mixed with a menadione solution (Sigma Aldrich Inc., St Louis, MO, USA) prepared with 1 mM acetone and with PBS containing 200 mM glucose ^13^.

Each strain was standardized at a concentration of 2.5x10 ^1^ cells/mL ^14^ and added to the wells of the 96-well culture plates (TPP®, Techno Plastic Products, Trasadingen, Switzerland) containing the media conditioned by the extracts. In the evaluation period (24h), the conditioned medium was removed from the wells of the culture plates ^14^ and they were washed twice in PBS by centrifugation in a plate rotor, at 2000 rpm for 2 min. Then, 200 µL of the XTT solution was added to each well. The culture plates were left on an orbital shaker at a speed of 75 rpm for 3h at 37°C, to allow the reaction in the XTT solution. After this period, the culture plate was centrifuged again at 10^º^C for 2 min, at a speed of 10,000 rpm to decant the cells and 200 µL of the supernatant from each well was transferred individually to a new 96-well plate ^12^ for the evaluation of cellular metabolism. For this, the content of each well was subjected to reading on a spectrophotometer (Synergy Mx Monochromator-Based Biotek®, Winooski, VT, USA) under the wavelength of 550 nm. As a positive control, sodium hypochlorite was used.

## Results

### Cytotoxicity analysis

**Figure 1:**
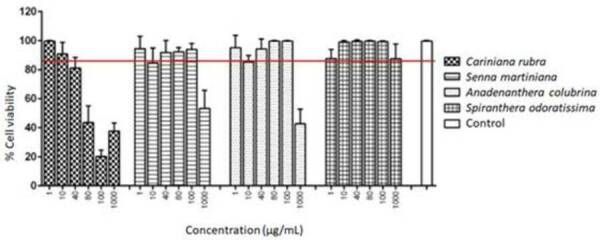
Percentage of viability of mouse fibroblasts in the presence of different concentrations of the tested extracts in vitro (*Cariniana rubra Gardiner ex Miers*, *Senna martiniana* H.S. Irwin & Barneby, *Anadenanthera colubrina* (*Vell*.) *Brenan* and *Spirantheraodoratissima*. The red line corresponds to 85% cell viability.

From the MTT colorimetric assay, it was possible to establish a quantitative index of cell viability of fibroblasts over 24 hours, in contact with the media conditioned by the extracts in concentrations of 1, 10, 40, 80, 100 and 1000 µg/mL (Figure 1).

Only the extracts of *Cariniana rubra* in concentrations of 40 µg/mL, 80 µg/mL, 100 µg/mL and 1000 µg/mL, *Senna martiniana* H.S. Irwin & Barneby in the concentration of 1000 µg/mL and *Anadenanthera colubrina* in the concentration of 1000 µg/mL were cytotoxic. These extracts in the other concentrations and the *Spiranthera odoratissima* extract did not present cytotoxicity. *Cariniana rubra Gardiner ex Miers* was the extract that showed the highest cytotoxicity.

The means of the original absorbance values at 550 nm obtained with the spectrophotometric readings of the experimental groups containing the concentrations of the extracts (*Carinianarubra Gardiner ex Miers*, *Senna martiniana* H.S. Irwin & Barneby, *Anadenanthera colubrina* (*Vell.*) *Brenan* and *Spiranthera odoratissima St. Hil*.) that presented values ≥85% of cell viability obtained through the MTT assay, in the tested incubation period (24h) are shown in Table 2.

**Table 2:** Percentages (%) of microbial metabolism inhibition after contact with the media conditioned by the extracts in different concentrations (µg/mL) in the evaluated incubation period (24h).

Extract	*C. albicans*	*E. coli*	*S. mutans*	*S. Aureus*	*A. actinomicetencomitans*	*E. faecalis*
*Cariniana rubra Gardinerex Miers*						
1	-	-	-	8.35	12.80	8.88
10	-	41.67	-	0.98	70.50	11.49
*Senna martiniana* H.S. Irwin & Barneby						
1	13.75	42.10	40.74	16.76	10.02	30.74
10	12.29	41.11	5.28	4.91	46.20	27.68
40	-	13.37	17.23	29.40	73.28	15.80
80	-	31.43	18.52	11.97	62.71	16.06
100	-	46.37	4.88	21.91	32.28	31.60
*Anadenanthera colubrina (Vell.) Brenan*						
1	-	52.20	22.51	4.79	41.93	26.90
10	-	43.53	23.21	20.13	79.22	57.31
40	-	55.33	15.64	17.37	80.52	35.38
80	-	48.22	25.60	13.07	70.13	43.47
100	-	59.60	-	-	63.45	54.05
*Spirantheraodoratissima St. Hil.*						
1	-	47.37	-	14.98	78.48	39.49
10	-	59.03	-	-	63.08	29.76
40	-	32.86	27.68	-	78.30	43.47
80	-	27.03	53.49	21.55	70.31	37.34
100	-	33.57	45.92	9.08	71.61	48.24
1000	-	37.27	-	-	48.61	4.96

### Evaluation antimicrobial effect of the extracts

As a positive control, sodium hypochlorite was used, causing 100% death of the microorganisms. In relation to *C. albicans*, *Senna martiniana* H.S. Irwin & Barneby was the only extract with antimicrobial action, with 13.75% inhibition occurring at a concentration of 1µg/mL; for *E. coli* the greatest inhibition (59.60%) was induced by the extract of *Carinniana rubra Gardiner ex Miers* at a concentration of 100 µg/mL; for *S. mutans*, the greatest inhibition was observed with the extract of *Spiranthera odoratissima St. Hil*. at a concentration of 80 µg/mL (53.49%); *S. aureus* showed a 29.40% inhibition in the concentration of 40 µg/mL of *Senna martiniana* H.S. Irwin & Barneby. The *A. actinomycetemcomitans* was highly inhibited by all extracts, with a higher percentage (80.52%) in contact with *Anadenanthera colubrina* (*Vell*.) *Brenan* extract at a concentration of 40 µg/mL. *E. faecalis* obtained greater inhibition (57.3%) in concentration 10 µg/mL also in contact with the extract of *Anadenanthera colubrina* (*Vell*.) *Brenan*. The extracts of *Cariniana rubra Gardiner ex Miers*, *Senna martiniana* H.S. Irwin & Barneby, *Anadenanthera colubrina* (*Vell*.) *Brenan* and *Spiranthera odoratissima St. Hil*.) presented acceptable cytotoxicity and antimicrobial action at different concentrations.

## Discussion

Based on the ISO 10993 standard, a material is considered cytotoxic when the results of the MTT test show values greater than 30% of the negative control (non-cytotoxic) ^15^. Within 24 hours, it was observed that cell viability in contact with the extracts remained close to the negative control, with the exception of *Cariniana rubra* extracts at concentrations of 40 µg/mL, 80 µg/mL, 100 µg/mL and 1000 µg/mL, *Senna martiniana* H.S. Irwin & Barneby at a concentration of 1000 µg/mL and *Anadenanthera colubrina* at a concentration of 1000 µg/mL.

Although there is no standard of concentration and level of inhibition acceptable for comparison of the results of the activity of the extracts when compared with standard antibiotics, since its active principles are not yet well known, de Araújo et al. ^16^ proposed a classification for plant materials based on the results of minimum inhibitory concentration (MIC), considering as: strong inhibition - MIC up to 500 µg/mL; moderate inhibition - MIC between 600 and 1500 µg/mL and as weak inhibition - MIC above 1600 µg/mL. On the other hand, Mohammed et al. ^17^ consider that all plants used in popular medicine that show the minimum inhibitory concentration (MIC) values below 8 µg/mL are designated as active.

By the criterion of Mohammed et al. ^17^, *Senna martiniana* H.S. Irwin & Barneby extract showed antimicrobial activity, since at a concentration of 1µg/mL it showed 13.75% *C. albicans* inhibition, 42.10% *E. coli*, 40.74% *S. mutans*, 16.76% *S. aureus*; 10.02% *A. actinomycetencomitans* and 30.74% *E. faecalis* inhibition. These results can be explained by the presence of tripertenes or even a glycosylated dianthrone, present in *Senna martiana* H.S. Irwin & Barneby, as anthraquinones and other phenolic compounds are characterized by their antioxidant and antimycotic properties ^7^ ^,^ ^11^. Thus, *Senna martiniana* H.S. Irwin & Barneby extract is promising for the development of products with antimicrobial action, mainly for the prevention or treatment of candidiasis and infections caused by *E. coli*.

In the evaluation of the extract of *Cariniana rubra Gardiner ex Miers*, antimicrobial activity in the MIC value of 1µg/mL is observed for the microorganisms *E. coli* (41.67%), *S. aureus* (8.35%), *A. actinomicetencomitans* (12.80%) and *E. faecalis* (8.88%). 

Chemical analysis of methanol extract obtained from C. rubra stem bark showed the presence of saponins, tannins, free steroids, flavonols and flavones ^19^. Tannins and saponosides have the ability to complex with steroids, which explains the antifungal and hypocholesterolemic action of these metabolites ^11^. Among the most cited activities for saponins, stand out the hemolytic, molluscicidal, anti-inflammatory, antifungal/anti-yeast, antibacterial/antimicrobial, antiparasitic, cytotoxic/anti-tumor, and finally, antiviral activities ^18^. This result contradicts a previous study in which the extract of *Cariniana rubra* had no action against *S. aureus* ATCC 25923, *E. faecalis* ATCC 29212 and other bacteria not used in this study, even at concentrations above 8µg/mL ^11^. Lima Neto et al. ^18^ observed an antifungal action of the extract of *Cariniana rubra* against *C. albicans* (MIC 62.5 µg/mL) and in relation to bacterial strains, the most sensitive was *S. aureus* with MIC of 250 µg/mL for the tested extract. Santos et al. ^19^ did not perform microbiological tests, but found good anti-inflammatory results for the same extract. The differences found in the studies may be related to the genetic constitution (genotypes) of plant populations in each region, which would also be responsible for changes in the components of medicinal plants. However, the environmental factors are the ones that cause significant variations in its components, and consequently in the biological activity of the metabolites, as verified in the comparison between samples collected in different regions of Brazil ^4^ ^,^ ^5^ ^,^ ^9^ ^,^ ^16^ ^,^ ^18^.

In tests with *Anadenanthera macrocarpa* (*Benth*) *Brenan* extract, antimicrobial action was obtained with MIC below 8µg/mL against *E. coli* (52.20%), *S. mutans* (22.51%), *S. aureus* (4.79%), *A. actinomycetencomitans* (41.93%) and *E. faecalis* (26.90%). By increasing the MIC, a greater inhibition of *A. actinomycetencomitans* and *E. faecalis* was obtained (MIC 40mg/mL = 80.52%, for *A. actinomycetencomitans* and MIC 10µg/mL = 57.31% for *E. faecalis*). This plant is also popularly used for toothache and showed 22.51% inhibition against *S. mutans*, the main bacterium related to tooth decay ^4^ ^,^ ^17^ and 57.31% for *E. faecalis*, one of the species most involved in endodontic treatment failures ^3^.

De Araujo ^16^ found no inhibitory effect of the extract and *Anadenanthera macrocarpa* (*Benth*) *Brenan* at the concentrations tested in *S. mutans*. The results by Lima et al. ^5^ found the antifungal potential of this plant, as the extract and its active fraction inhibited the growth of *C. albicans* (MIC = 0.31µg/mL), demonstrating strong antimicrobial activity, opposite to this research, which did not result in inhibition of metabolism in contact with the extract. Lima Neto et al. ^18^ evaluated antimicrobial action against *S. aureus*, obtaining a positive result/sensitivity with MIC of 250 µg/mL, without mentioning the percentage of inhibition.

In the research by Barreto et al. ^20^ natural products based on *Anadenanthera* did not have direct inhibitory activity at tested clinically relevant concentrations, however, natural products obtained from this extract are described as aminoglycoside activity intensifiers against a methicillin-resistant strain of *S. aureus* ^20^.

The extract of *Spiranthera odoratissima St. Hil*. is considered inactive only for the fungus *C. albicans*, corroborating a previous study ^21^. However, it obtained values of minimum inhibitory concentration (MIC) lower than 8 mg/mL in all other microorganisms tested, being more effective against *A. actinomycetencomitans*, with MIC 1 µg/mL it obtained 78.48% of microbial metabolism inhibition. No research was found with the other microorganisms and the tested extract. Phenols, tannins, coumarins, flavonoids, triterpenes/sterols, anthraquinones and anthocyanins were detected in the phytochemical screening of the leaves, in addition to alkaloids, coumarins, saponins and starch were detected in the roots ^21^, which corroborates the results obtained in this investigation. In other studies, an anxiolytic effect and anti-inflammatory action were observed ^22^, in addition to anti-Leishmania activity in vitro ^23^, although this extract is popularly used to treat rheumatism ^10^ ^,^ ^22^.

The results, in general, of a slight reduction in cell viability and microorganism death have been reported in previous works ^4^ ^,^ ^5^ ^,^ ^9^. These effects are probably, as discussed by other authors, most likely due to the components identified in these extracts as: flavonoids (quercitin, gallic acid, etc.) and tannins ^9^ ^,^ ^16^ ^,^ ^18^.

This work was the first to demonstrate the antimicrobial action of *Spiranthera odoratissima St. Hil*. against the different microorganisms studied. *A. actinomycetencomitans*, the main inhibited microorganism, has been proposed as a link between periodontitis and autoimmunity in rheumatoid arthritis (RA) due to its ability to induce citrullinated autoantigens directed by anti-citrullinated protein antibodies ^24^. Thus, the action of *Spiranthera odoratissima St. Hil*. against this bacterium demonstrated in this study corroborates its popular use for the treatment of rheumatoid arthritis as well as for periodontitis.

The results presented show the possibility of developing new antifungal agents based on *Senna martiniana* and new products, such as mouthwashes and dental materials, based on *Anadenanthera colubrina* (*Vell*.) *Brenan* (angico), which inhibited the metabolism of all tested bacteria, with a high percentage (80.52%) for *A. actinomycetencomitans*, a microorganism that is strongly associated with aggressive forms of periodontitis ^3^. All extracts proved to be promising for the inhibition of pathogenic microorganisms studied.

The result of this microbiological examination encourages further investigation to find and develop natural components with antimicrobial activity and against oral pathogens. Due to the increased incidence of multidrug-resistant bacteria, despite the use of oral antiseptics, the application of herbal extracts can be an effective alternative treatment strategy against oral pathogens. Interestingly, the various side effects of conventional oral care products, such as allergies, intolerable taste, tooth coloring, toxicity and antimicrobial resistance, triggered the search for alternative antimicrobials, at best in natural circumstances.

Cytotoxicity tests are recommended for all materials used in the health field, and in vitro study models are an essential alternative in the search for cytotoxicity of dental materials today. These tests allow a quick evaluation, improve standardized protocols, produce quantitative and comparable data, and due to their sensitivity, they allow toxic materials to be discarded prior to animal experiments ^25^. However, the methodology has its limitations and shall be interpreted as preliminary results.

Despite the merit of the *in vitro* test, it cannot be said that the material is biocompatible, since the *in vitro* cytotoxicity test is the first step in analyzing the material under study. On the other hand, a positive cytotoxicity test can be a sign that the material contains one or more substances that may be of clinical importance ^25^. 

The development of natural resources is crucial for developing countries, contributing to economic growth and improving people's health at low cost. Thus, combinations of these plant extracts could serve as the main antimicrobial components in alternative antibacterial formulations, facilitating the prevention of related oral diseases biofilm, such as caries or periodontitis ^4^.

Future researches should propose more elaborate experiments with the compounds that showed the most promising results. It is suggested that the extracts be applied in future *in vivo* research for the development of mouthwashes and dental materials to treat the different pathologies caused by the studied microorganisms.

Considering the proposed methodology and the limitations of the study, it is reasonable to conclude that the analyzed extracts of *Cariniana rubra Gardiner ex Miers*, *Senna martiniana* H.S. Irwin & Barneby, *Anadenanthera colubrina* (*Vell.*) *Brenan* and *Spiranthera odoratissima St. Hil*. showed acceptable cytotoxicity at different concentrations and were promising for inhibition of the pathogenic microorganisms studied.
